# Case Report: Overlap Between Long COVID and Functional Neurological Disorders

**DOI:** 10.3389/fneur.2021.811276

**Published:** 2022-01-28

**Authors:** Luana Gilio, Giovanni Galifi, Diego Centonze, Mario Stampanoni Bassi

**Affiliations:** ^1^Istituto di Ricovero e Cura a Carattere Scientifico (IRCCS) Neuromed, Pozzilli, Italy; ^2^Department of Systems Medicine, Tor Vergata University, Rome, Italy

**Keywords:** COVID-19, functional neurological disorders, post-acute COVID, long COVID, case report

## Abstract

Long lasting symptoms have been reported in a considerable proportion of patients after a severe acute respiratory syndrome Coronavirus 2 (SARS-CoV-2) infection. This condition, defined as either “post-acute coronavirus disease (COVID),” “long COVID,” or “long-haul COVID,” has also been described in outpatients and in individuals who are asymptomatic during the acute infection. A possible overlap exists between this condition and the functional neurological disorders (FNDs). We report a 23-year-old man who developed, after asymptomatic COVID-19, a complex symptomatology characterized by fatigue, episodic shortness of breath, nocturnal tachycardia, and chest pain. He also complained of attention and memory difficulties, fluctuating limb dysesthesia, and weakness of his left arm. After neurological examination, a diagnosis of FND was made. Notably, the patient was also evaluated at a post-COVID center and received a diagnosis of long COVID-19 syndrome. After 4 months of psychoanalytic psychotherapy and targeted physical therapy in our center for FNDs, dysesthesia and motor symptoms had resolved, and the subjective cognitive complaints had improved significantly. However, the patient had not fully recovered as mild symptoms persisted limiting physical activities. Long-term post COVID symptoms and FNDs may share underlying biological mechanisms, such as stress and inflammation. Our case suggests that functional symptoms may coexist with the long COVID symptoms and may improve with targeted interventions. In patients presenting with new fluctuating symptoms after SARS-CoV-2 infection, the diagnosis of FNDs should be considered, and the positive clinical signs should be carefully investigated.

## Introduction

The severe acute respiratory syndrome Coronavirus 2 (SARS-CoV-2) has been associated with a broad range of clinical manifestations including fever, respiratory, cardiovascular, gastrointestinal, and neurological symptoms during the acute phase of the disease (1). Long lasting symptoms, such as fatigue, dyspnea, cognitive dysfunction, and sleep disorders have been described in a considerable proportion of patients who suffered from Coronavirus disease 2019 (COVID-19) (2). This condition, defined as “post-acute COVID,” “long COVID,” or “long-haul COVID,” negatively affects the quality of life and often requires additional clinical assistance. Notably, long term sequelae have been reported not only in patients who are previously hospitalized in intensive care units, but also in non-hospitalized patients with mild symptoms during the acute infection, as well as in asymptomatic patients (3). Considerable effort is being made to better understand the pathogenesis of post-COVID symptoms. While it is important to monitor the long-term effects of SARS-CoV-2, alternative factors should be considered, among them is the functional neurological disorders (FNDs).

## Case Presentation

Herewith, we report a 23-year-old man who contracted COVID-19 in February 2021. The diagnosis was made by rRT-PCR on a nasopharyngeal swab performed after contact with an infected individual. The patient was asymptomatic at the time of test positivity and started a period of quarantine. He was alone, abroad for study reasons, and the notification of the infection, together with self-isolation, caused an intense stress. In the following days he began to complain of fatigue, episodic shortness of breath, nocturnal tachycardia, and chest pain; an electrocardiogram and chest x-ray were negative. After his return to Italy, the symptoms persisted. In addition, he developed attention and memory difficulties, and a fluctuating limb dysesthesia. Physical examination and an extensive diagnostic work-up including Holter ECG, echocardiogram, and chest CT were within normal limits. In April 2021, the patient underwent his first neurological examination, reporting weakness and clumsiness of his left arm. He was diagnosed with a functional movement disorder as his complaints were variable and distractible and not compatible with an organic neurological disorder (brain MRI, nerve conduction studies, electromyography, and evoked potentials were negative). Arm weakness was characterized by extreme slowness and drift without pronation, and deep tendon reflexes were normal. Importantly, weakness has been reported as one of the most common functional motor symptoms, being frequently associated with non-motor disturbances such as anxiety and fatigue (4). The patient was then referred to our center for FNDs. The patient had no history of psychiatric disturbances or mood disorders, and no pathological personality traits were identified. Stress related to social expectations and isolation, along with health concerns related to SARS-CoV-2 infection may be considered as precipitating factors. Neuropsychological evaluation showed a normal cognitive profile, presence of depression, and elevated anxiety levels. After carefully discussing the diagnosis of FND with the neurologist, a course of psychoanalytic psychotherapy and targeted physical therapy was planned (5). Notably, a few weeks later, the patient was evaluated at a post-COVID center of another hospital. After an additional diagnostic assessment with negative results, he received a diagnosis of a post-COVID-19 syndrome.

After 4 months, dysesthesia and motor symptoms had resolved, and the subjective cognitive complaints had improved significantly. The patient returned to his studies and social activities but had not fully resumed the physical activity, as post-exertional malaise and chest pain, and a fluctuating muscle tension in his back and left arm persisted.

## Discussion

Patients presenting with new symptoms after COVID-19, whether asymptomatic during the acute infection, are currently referred to post-COVID centers. New signs and symptoms that appear during or after SARS-CoV-2 infection, that persist for at least 2 months even if fluctuating or relapsing, and were not explained by an alternative diagnosis, meet the criteria for post-COVID-19 syndrome (2). A broad spectrum of symptoms has been reported including shortness of breath, palpitations, chronic fatigue, pain, motor, and sensory deficits. It is worth noticing that this new clinical entity may have some overlap with the FNDs.

According to the DSM-5, FNDs are characterized by one or more neurologic symptoms that show incompatibility with established neurological or medical disorders (6). Therefore, the diagnosis of FNDs is based on positive clinical signs suggestive of a functional basis and on the exclusion of organic disease (6). The presence of psychiatric or mood disorders is not required, although it is generally accepted that both play a role as predisposing factors.

The COVID-19 pandemic profoundly influenced the lifestyle in both healthy subjects and patients with neurological conditions, and has been associated with psychiatric features, sleep disorders, and worsening of neuromuscular symptoms (7–9). The COVID-19 pandemic produced a significant increase in psychiatric symptoms, including anxiety, depression, and post-traumatic stress disorder (10). In addition to health concerns, difficulties in social, family, and work relationships represented a source of stress, made even worse by periods of self-isolation and lockdown.

As expected, the emergence of functional neurological symptoms, such as tremor and tic-like behaviors were evident, after COVID-19 has been described (11, 12). Similarly, in patients with psychogenic non-epileptic seizures (PNESs), COVID-19 pandemic influenced the characteristics of functional seizures (13). In these patients, often affected by mild symptoms in the acute phase, distinctive clinical features (i.e., positive clinical signs), along with an association with mood disorders and psychosocial stressors, have been reported (12, 13). These findings suggest that COVID-19 pandemic may favor the emergence of FNDs, in line with the hypothesis that stress represents a crucial precipitating factor. Furthermore, these studies confirm that FNDs can be effectively diagnosed using DSM-5 criteria, particularly in the presence of positive clinical signs, although diagnosis may be difficult in patients with pain or sensory deficits (6). In addition, it has been reported that traumatic injuries often precede the onset of FNDs (4); thus, symptomatic COVID-19 infection may act as a trigger in some patients, while also increasing the attention to physical sensations.

The pathogenesis of neurologic symptoms related to SARS-CoV-2 infection is complex, and multiple mechanisms have been hypothesized, including direct toxic effects, autoimmune activation, and inflammation (14). While some symptoms may be ascribed to a local effect of the virus (i.e., anosmia due to damage of the olfactory nerve), others, as myoclonus, opsoclonus, and ataxia, could have an autoimmune etiology (14). Notably, cases of Guillan-Barré syndrome have also been reported after COVID-19 (15). Other long-term neurological symptoms including non-specific attention and memory complaints, fatigue, headache, and dizziness may arise from the inflammatory response to the virus, characterized by the release of proinflammatory molecules (16). In several clinical conditions, peripheral and central inflammations have been associated with behavioral alterations and mood disorders. Therefore, in the acute and subacute phases of COVID-19, inflammation may contribute to mood disorders, and indirectly promote the occurrence of FNDs.

## Conclusion

Coronavirus disease 2019 (COVID-19), even the asymptomatic type, represents a stressor that may act in some individuals as a precipitating factor for FNDs. Functional symptoms may coexist with long COVID symptoms and can improve with targeted interventions. In patients presenting with new fluctuating symptoms after SARS-CoV-2 infection, the diagnosis of FNDs should be considered, and positive clinical signs should be carefully investigated. Long-term post-COVID symptoms and FNDs may share underlying biological mechanisms, such as stress and inflammation.

## Data Availability Statement

The raw data supporting the conclusions of this article will be made available by the authors, without undue reservation.

## Ethics Statement

Ethical review and approval was not required for the study on human participants in accordance with the local legislation and institutional requirements. The patients/participants provided their written informed consent to participate in this study. Written informed consent was obtained from the individual(s) for the publication of any potentially identifiable images or data included in this article.

## Author Contributions

All authors were involved in the analysis and interpretation of findings, they proved the manuscript, contributed for important intellectual content, and contributed to writing and approved the final manuscript.

## Funding

The study was supported by the Italian Ministry of Health (Ricerca corrente and 5 per mille public funding to IRCCS Neuromed).

## Conflict of Interest

The authors declare that the research was conducted in the absence of any commercial or financial relationships that could be construed as a potential conflict of interest.

## Publisher's Note

All claims expressed in this article are solely those of the authors and do not necessarily represent those of their affiliated organizations, or those of the publisher, the editors and the reviewers. Any product that may be evaluated in this article, or claim that may be made by its manufacturer, is not guaranteed or endorsed by the publisher.
